# Evaluation of Coagulation Profile Alterations in Patients Undergoing Laparoscopic Cholecystectomy With Carbon Dioxide (CO₂) Pneumoperitoneum

**DOI:** 10.7759/cureus.94160

**Published:** 2025-10-08

**Authors:** Aravind Kumar, Santhaseelan R G, Barathi Raja Kuppusami, Samir Ahmad

**Affiliations:** 1 General Surgery, Sree Balaji Medical College and Hospital, Chennai, IND

**Keywords:** cholecystectomy, coagulation profile, d-dimer, international normalized ratio, laparotomy, pneumoperitoneum, prothrombin time

## Abstract

Background and aim: Laparoscopic cholecystectomy is commonly performed for the removal of the gallbladder. This widely performed minimally invasive surgical procedure requires the creation of a pneumoperitoneum by insufflation of carbon dioxide (CO₂), which may affect coagulation dynamics. The objective was to assess the impact of CO₂ pneumoperitoneum on coagulation parameters in patients undergoing elective laparoscopic cholecystectomy.

Methods: All patients undergoing elective laparoscopic cholecystectomy, aged between 18 and 60 years, were included in this prospective observational study. Post-operative samples were collected 6 h after the creation of pneumoperitoneum. The patients’ blood samples were collected pre- and post-surgery and analyzed for D-dimer, blood coagulation profile, and international normalized ratio (INR).

Results: A total of 81 patients were enrolled in this research. A male dominance (59 {72.8%}) was noted with a male-to-female ratio of 2.7:1, with a mean age of 38.9±11.9 years. A decreased trend was observed in post-operative values of prothrombin time (PT) (pre-operative: 12.0±0.448 and post-operative: 11.8±0.458 s) and activated partial thromboplastin time (aPTT) (pre-operative: 30.0±2.13 and post-operative: 28.0±2.22 s), with ~1 s and 2 s, respectively. These changes were indicative of a procoagulant state. The INR (0.998±0.0379) and D-dimer (0.868±0.314) values were noted to increase significantly post-operatively.

Conclusion: Laparoscopic cholecystectomy with CO₂ pneumoperitoneum creates a mild, subclinical hypercoagulable state marked by shortened clotting times and elevated fibrin degradation products. This state rarely causes symptomatic thrombosis in low-risk patients but warrants vigilance and prophylaxis in those with additional risk factors.

## Introduction

Gallstone disease, the leading indication for cholecystectomy, is a common global health problem. A recent meta-analysis estimates that roughly 6% of the world’s population harbors gallstones, with higher prevalence in females than males. There is marked geographic variation; in Asian countries, gallstone prevalence is relatively low, whereas Western nations report significantly higher rates, ranging from 20-25% in certain populations [1]. India, in particular, has a reported cholelithiasis prevalence of around 4-6%, with a greater burden observed in North Indian communities compared to the South Indian communities [2]. Cholecystectomy is one of the most frequently performed surgical procedures worldwide. Laparoscopic techniques account for the vast majority of these. India also conducts a substantial number of gallbladder removals each year, although exact national figures are not documented [3]. Laparoscopic cholecystectomy differs from other common laparoscopic procedures in many important physiological aspects. Majorly, the venous return from the lower limbs is reduced, enhancing the venous stasis. A longer duration can prolong exposure to the elevated intra-abdominal pressure and CO₂ absorption. Additionally, the surgical field in laparoscopic cholecystectomy is located close to the diaphragm and major vessels, making hemodynamic changes from pneumoperitoneum more pronounced. Together, these factors create a stronger predisposition to subtle hypercoagulability in laparoscopic cholecystectomy patients than in other routine laparoscopic procedures.

Laparoscopic cholecystectomy is recommended for managing symptomatic gallbladder disease globally. Compared to open surgery, laparoscopic cholecystectomy (LC) was found to have less post-operative pain with faster recovery and shorter hospitalization durations. The procedure involves the creation of a carbon dioxide (CO₂) pneumoperitoneum, insufflating the abdomen with CO₂ gas to elevate the abdominal wall and provide operative space. This pneumoperitoneum, typically at pressures of 10-15 mmHg, and the patient's head-up reverse Trendelenburg position significantly alter normal physiology [3,4]. CO₂ absorption can cause hypercapnia and respiratory acidosis, while increased intra-abdominal pressure reduces venous return, elevates systemic vascular resistance, and may induce splanchnic circulatory changes. These hemodynamic stresses, alongside surgical trauma, have potential prothrombotic effects. Indeed, it is recognized that decreased venous return from the legs and a state of relative hypercoagulability during CO₂ pneumoperitoneum may together create a risk factor for deep vein thrombosis (DVT) in LC patients [5,6]. However, the precise impacts of CO₂​​​​​​​ pneumoperitoneum on the human coagulation system remain an area of ongoing investigation. An elevated intra-abdominal pressure can compress the inferior vena cava and iliac veins, leading to venous stasis in the lower extremities [4]. Simultaneously, absorbed CO₂​​​​​​​ and peritoneal stretch may stimulate catecholamine release and endothelial activation. It has been hypothesized that pneumoperitoneum may even cause subtle injury or activation of the vascular endothelium, promoting the initiation of the coagulation cascade in a manner analogous to surgical trauma [6,7]. LC is minimally invasive on the surface; it has been associated with procoagulant triggers. However, there is contrasting evidence present [4,5]. It highlights a gap in understanding the association and raises a need to investigate patient-specific or regional factors that might modulate the hemostatic response to CO₂​​​​​​​ pneumoperitoneum. Attributed to the widespread use of LC and the potentially serious consequences of thromboembolism, this study aimed to investigate the association between CO₂ pneumoperitoneum and the coagulation profile of patients undergoing LC, as well as the risk assessment of post-operative thrombosis in the study group. It included identifying risk factors and developing an understanding of prophylactic measures in at-risk patients.

## Materials and methods

Study design and setting

This prospective observational study was conducted in the Department of General Surgery at Sree Balaji Medical College and Hospital, Chennai, India, over an 18-month period from April 2023 to October 2024.

Sample size and sampling method

The sample size was determined using the formula for paired comparisons, based on expected changes in coagulation parameters. The final sample size was calculated to be 81 patients, ensuring adequate statistical power (1-β) and a significance level of 5%. Purposive sampling was used to select eligible patients based on predefined inclusion and exclusion criteria.

Study population

A total of 81 patients with indications for gallstones, gallbladder polyps, or chronic cholecystitis, aged between 18 and 60 years, and scheduled for elective laparoscopic cholecystectomy, and willing to provide consent, were enrolled for this research. The surgery duration, which was included in this research, ranged from 90 to 180 min. Exclusion criteria were marked as a known history of bleeding disorders, coagulopathy, or anticoagulant therapy, pre-existing deep vein thrombosis or associated hypertension, pregnancy or known malignancy, conversion to open cholecystectomy, and a surgery duration of >180 min. Patients who were unwilling to consent were also excluded.

Data collection procedure

A detailed history and clinical examination data were recorded in a predefined questionnaire. Basic laboratory investigations were performed, and baseline blood samples were collected before the induction of pneumoperitoneum. Post-operative samples were collected 6 h after the creation of pneumoperitoneum. The patients’ blood samples were analyzed for prothrombin time (PT), activated partial thromboplastin time (aPTT), international normalized ratio (INR), and D-dimer levels. All blood samples were drawn according to standard guidelines with a duplex ultrasound scan in high-risk patients for deep vein thrombosis (DVT).

Pre-operative and post-operative assessment

All patients underwent detailed pre-operative clinical evaluation, including history, physical examination, and routine investigations. Baseline coagulation profile, comprising PT, aPTT, INR, and D-dimer levels, was obtained immediately before surgery. Each patient was assessed for risk of DVT using a standard risk scoring system. Post-operative coagulation parameters were repeated 24 h after surgery. PT and INR were measured via the prothrombin time method using thromboplastin reagents standardized to the International Sensitivity Index (ISI). The aPTT was evaluated using the kaolin-activated clotting time methodology. D-dimer levels were quantified using an immunoturbidimetric assay and reported in micrograms per milliliter (µg/mL).

Surgical procedure and outcomes

All patients underwent standard four-port laparoscopic cholecystectomy under general anesthesia. CO₂ pneumoperitoneum was maintained at an intra-abdominal pressure of 12 mmHg throughout the procedure. Reverse Trendelenburg positioning was employed routinely to facilitate gallbladder visualization as per standard guidelines. Primary outcomes included changes in coagulation parameters (PT, aPTT, INR, and D-dimer) between pre-operative and post-operative values. The secondary outcome was the incidence of post-operative DVT. The patients were followed up three months post-operatively.

DVT surveillance

All patients were clinically evaluated for signs of DVT in the post-operative period, including lower limb swelling, tenderness, or calf pain. In cases of clinical suspicion, duplex ultrasonography was performed. All the recorded data were analyzed using SPSS version 26.0 (Armonk, NY: IBM Corp.). Continuous variables, such as PT, aPTT, INR, D-dimer levels, age, and duration of surgery, were summarized as mean±standard deviation. Categorical variables, such as gender, BMI category, and DVT risk classification, were presented as frequencies and percentages. The normality of data distribution was assessed using the Shapiro-Wilk test, and a paired sample t-test was employed to compare the pre-operative and post-operative values of each coagulation parameter. A two-tailed significance level of 5% (p<0.05) was considered statistically significant.

## Results

A total of 81 participants were enrolled in this research after screening for all the inclusion and exclusion criteria. The male-to-female ratio observed in the study group was 2.7:1. The majority of the study participants were ≤50 years of age. The rest of the demographic profile of the study population is presented in Table 1.

**Table 1:** Demographic profile of the study group. DVT: deep vein thrombosis

Parameters	Value, n (%)/mean±SD
Total participants	81 (100)
Male	59 (72.8%)
Female	22 (27.2%)
Age (years)	38.9±11.9
Age (<30)	22 (27.2%)
Age (31-40)	22 (27.2%)
Age (41-50)	20 (24.7%)
Age (51-60)	17 (21.0%)
BMI (kg/m²) (normal; range: 18.5-24.9)	41 (50.6%)
BMI (kg/m²) (overweight; range: 25-29.9)	23 (28.4%)
BMI (kg/m²) (obese: >30)	17 (21.0%)
DVT risk	3 (3.7%)
Surgery duration (min)	135±27.2

A comparative evaluation of the coagulation profile showed a statistically significantly lower value of PT and aPTT, with significantly higher values of D-dimer and INR observed in this research. Mean pre-operative and post-operative PT values were within normal limits; however, they were found to be significant (T=9.26, p<0.001). This reduction in clotting time was indicative of increased coagulation risk post-surgery. The pre-operative and post-operative aPTT values were also within the normal range of 25-35 s but noted to be decreased significantly (T=32.6, p<0.001), in similarity to the PT values, suggestive of a hypercoagulable shift with reduced activity in the intrinsic and common coagulation pathways. The changes in INR levels were statistically significant, but due to the small sample size, this needs to be validated with a larger study cohort. Clinically, elevated D-dimer post-surgery may suggest a hypercoagulable state, common after surgical trauma. D-dimer increased substantially (T=-22.3, p<0.001), indicating enhanced clot breakdown and fibrinolysis, possibly as a response to tissue injury or thrombus formation in post-surgical trauma. One patient was found to have DVT and was managed appropriately. These changes are clinically relevant, especially in monitoring post-operative thrombotic risks. The comparative assessment of the pre-operative and post-operative blood marker profiles of the study participants is presented in Table 2.

**Table 2:** Comparative assessment of pre-operative and post-operative coagulation profile. *Paired sample t-test. PT: prothrombin time; aPTT: activated partial thromboplastin time; INR: international normalized ratio; FEU: fibrinogen equivalent unit

Blood parameters	Reference range	Pre-operative value (mean±SD)	Post-operative value (mean±SD)	T-statistic	p-Value*
PT	11-13.5 s	12.0±0.448	11.8±0.458	9.26	<0.001
aPTT	25-35 s	30.0±2.13	28.0±2.22	32.6	<0.001
INR	0.8-1.2	0.997±0.0375	0.998±0.0379	7.81	<0.001
D-dimer	0.5 µg/mL FEU	0.306±0.0996	0.868±0.314	-22.3	<0.001

## Discussion

A total of 81 patients undergoing elective LC were evaluated for changes in standard coagulation parameters after surgery. The male-to-female ratio was found to be higher (2.7:1) in this study cohort. This gender ratio difference is not in alignment with published literature, which suggests female predominance in gallstone incidence. This can be attributed to referral bias, as the study was conducted in a tertiary care referral center, where sociocultural and healthcare-seeking behaviors in our population may influence the presentation and management of conditions, particularly among females, who might present later or be managed conservatively compared to males. Moreover, a higher prevalence of risk factors, such as alcohol and tobacco consumption, and metabolic comorbidities in males, can be an additional contributing reason. Post-operatively, there was a small but statistically significant shortening of PT, from a mean of 12.0 to 11.8 s, and aPTT from a mean of 30.0 to 28.0 s compared to pre-operative values, indicating a shift toward a procoagulant state. The INR showed a very slight decrease from 0.997 to 0.988 on average, essentially remaining within normal limits. Meanwhile, D-dimer levels increased sharply, approximately threefold higher than baseline, by 24 h after surgery, signaling activation of coagulation and fibrinolysis. Importantly, no patient developed DVT in the post-operative period, a finding consistent with the low thromboembolic risk generally associated with LC in low-risk individuals.

Prothrombin time

PT is inherently an insensitive marker for hypercoagulability; a very prothrombotic state might shorten it slightly, but minor coagulation activation can still occur without tipping PT beyond normal limits. The average PT decreased slightly after laparoscopic cholecystectomy from 12.0±0.45 s pre-operatively to 11.8±0.46 s post-operatively, which was significant with a p<0.001. Although the difference was only 0.2 s, it was statistically significant owing to the sample size and low variability. It was suggestive of a trend toward faster blood clotting. A shortened PT indicates a marginally hypercoagulable shift in the extrinsic pathway in the immediate post-operative period. This finding aligns with several reports in the literature. An observational study by Buhe et al. from China noted that mean PT had shortened by about 0.5 s compared to baseline 8 h post-LC procedure [8]. Similarly, a recent Indian study involving 113 LC patients reported a significant post-operative PT decrease with a mean reduction of 0.16 s, corroborating that pneumoperitoneum induces a mild hypercoagulable state [9]. These findings strengthen the evidence that laparoscopic surgery can subtly accelerate clotting on standard PT assays. However, contrasting evidence is indicated by a 2009 research study, which observed no statistically significant change in PT after LC [10]. A recent study by Singh et al. also found no significant difference in baseline and 24 h post-operative PT levels [4].

A few studies have documented an increase in PT after LC, contrary to the findings of this study. Ntourakis et al. in Greece noted that the mean PT rose from 1.04 pre-operative to 1.12 post-operative in 119 patients [11]. Similarly, Donmez et al. found in a randomized trial that PT was slightly prolonged 1 h after surgery under both low-pressure and standard-pressure pneumoperitoneum; in the 14 mmHg group, for instance, mean PT increased from 12.4 s to 13.4 s at 24 h. Despite PT/INR prolongation, fibrinogen levels were elevated post-operatively. D-dimer was significantly elevated, implying active coagulation with some factor consumption or dilution effect. Thus, a slight PT increase post-LC may paradoxically occur in individuals who mounted a strong coagulation response, leading to consumption of factor VII, which predominantly affects PT/INR. A drop of factor VII activity was observed in LC post-operative patients developing DVT, which was suggestive of the potential of utilization of PT as a marker of coagulation [12]. Importantly, research studies still observed concurrent rises in procoagulant factors and D-dimer, indicating that a prolonged PT did not signify a hypocoagulable state, but rather a consumption of clotting factors amid overall hypercoagulation [13,14]. The occasional reports of PT prolongation likely reflect extreme cases of coagulation activation or methodological differences. Clinically, since PT changes are modest and usually within normal range, they have not been associated with bleeding complications in LC patients. Monitoring PT/INR trends can provide insights into the balance between coagulation and consumption. This research observed that the slight PT decrease reinforces that CO₂ pneumoperitoneum and surgical stress tilt the hemostatic balance toward faster clot formation, although not enough to breach normal PT limits.

Activated partial thromboplastin time

A post-procedural aPTT shortening was observed with mean values decreasing from 30.0±2.1 s pre-operatively to 28.0±2.2 s post-operatively, which was significant with p<0.001. This reduction in aPTT indicates an accelerated intrinsic pathway activity post-LC, consistent with hypercoagulability. The post-operative aPTT shortening suggests that blood clotting initiated via the contact pathway occurs more quickly after pneumoperitoneum, likely due to elevated levels of clotting factors or other procoagulant changes triggered by surgery. The findings of this research are in line with multiple studies that have documented similar aPTT changes as reported by Singh et al*.*, Garg et al*.*, and Buhe et al., with a range of 1-4 s [4,5,8]. These findings across diverse settings underscore that intrinsic pathway clotting times tend to shorten after laparoscopic gallbladder surgery, reflecting a generalized prothrombotic response. It should be noted that not every study finds a statistically significant aPTT change, and some have reported divergent results under certain conditions. Natkaniec et al. in Poland observed minimal change in coagulation times in their laparoscopic surgery patients; aPTT was essentially unaltered alongside an unchanged PT/INR [15]. Methodological differences and patient factors can contribute to such variability. The majority of evidence supports a post-operative aPTT shortening in LC [16]. The results of this research concur with the intrinsic pathway being moderately activated post-LC. The shortened aPTT likely reflects elevated levels of intrinsic clotting factors (VIII, IX, XI) and possibly reduced anticoagulant activity immediately after surgery. Importantly, even though these aPTT changes were statistically significant, they left values within normal ranges for most patients, implying a subclinical hypercoagulability rather than an overt coagulopathy [11]. In patients with additional risk factors, such as acceleration of clotting, it could contribute to thrombosis. In summary, the intrinsic clotting pathway tends to become more active after LC, as evidenced by aPTT kinetics, a finding echoed by multiple Indian and international studies and exemplified in this study.

International normalized ratio

INR remains stable mainly in the context of laparoscopic cholecystectomy for most patients. INR, being a standardized derivative of PT, mirrored the PT findings with only a trivial post-operative change. The mean INR decreased from 0.997 pre-operative to 0.988, which was statistically significant. Clinically, INR essentially remained 1.0, which was within normal limits both pre- and post-operatively. Thus, LC did not induce any clinically substantial alteration in the prothrombin ratio, which was consistent with most literature, which reports that INR is essentially unchanged after laparoscopic cholecystectomy. Minor variations could result from hemodilution or assay differences, but importantly, no patient developed an INR outside the normal range post-operatively. This is reassuring, as it means LC did not cause any coagulopathic tendency in normal-risk individuals. Mechanistically, a drop in INR could result from hemoconcentration or increased factor levels due to surgical stress. Additionally, reduced plasma volume immediately after surgery might transiently concentrate clotting factors, slightly lowering INR [9,11,12]. Post-operatively, routine laboratory parameters are mostly within normal limits in LC patients, which is presumed to have low thrombosis risk. However, it should be paired with more sensitive markers or perform serial measurements, which can help appreciate the underlying hypercoagulable shifts [11,17]. Hence, the INR in this study group did not raise concern on its own; it must be interpreted alongside other findings to gain meaningful insights into post-operative coagulation changes.

D-dimer

D-dimer, a fibrin degradation product, is a key marker of coagulation and fibrinolysis activation. D-dimer levels surged post-operatively, rising from a mean of 0.306 mg/L pre-operative to 0.868 mg/L post-operatively in this study group. This significant elevation in D-dimer strongly indicated that laparoscopic cholecystectomy triggered substantial fibrin formation and breakdown, even though clinically overt thrombosis did not occur. D-dimer was the most changed parameter among all the study parameters. Multiple studies have reported post-LC D-dimer elevations, reflecting subclinical clotting. Ntourakis et al. found that D-dimer levels rose from 0.38 mg/L pre-operatively to 0.90 mg/L at 24 hours post-operatively. These findings underline that even low-risk patients develop fibrin clots that are subsequently lysed, releasing D-dimer [11]. It is noted that D-dimer was already elevated at the end of pneumoperitoneum and continued to climb in the first hour after surgery. The authors emphasized that the first 8 h post-LC is a period of hypercoagulability with active fibrin turnover, which aligns with our timing of measurement at 24 h post-operatively [8]. An Indian study by Tudu et al. reported that D-dimer levels post-LC were about three times higher than pre-operative levels [9]. D-dimer was instructive because it shows a dose-response as follows: higher intra-abdominal pressure led to higher D-dimer, suggesting more fibrin formation under greater venous stasis. The magnitude of the D-dimer increase in LC patients post-procedure is clinically meaningful, although still generally below levels associated with overt thrombosis in medical patients [12]. It signals that fibrin clots were forming intra- or post-operatively and undergoing lysis. These could be microthrombi in the surgical field or peripheral veins that never grew large enough to cause symptoms. An important point is that no patients had symptomatic DVT or pulmonary embolism, so the D-dimer rise represents subclinical activity. However, there is rare contradictory evidence reporting a decrease in D-dimer levels [5,14]. A post-operative D-dimer rise might not necessitate intervention in low-risk cases, but it does highlight a window of risk with recommendations suggesting vigilance in the immediate post-operative period, given the high risk of thrombosis.

Post-operative DVT incidence

It is essential to acknowledge that DVT can occur after LC, though uncommonly, and is more likely in higher-risk individuals or if sought by sensitive screening. A study by Milic et al. systematically screened patients with duplex ultrasound and found a DVT incidence of 6.9% in laparoscopic cholecystectomy patients [14]. Most of those thrombi were confined to calf veins and were asymptomatic, detected only by active surveillance. It is indicative that subclinical thromboses in distal veins can be formed even during the short peri-operative period of LC, though they rarely progress to cause symptoms [14,18,19]. Extensive database reviews and guidelines support our findings. Historical data indicate that symptomatic pulmonary embolism after LC occurs in roughly 0.1-0.2% of cases [8]. The American Society of Hematology guidelines for venous thromboembolism (VTE) prophylaxis suggest against routine pharmacologic prophylaxis in patients undergoing laparoscopic cholecystectomy who have no additional risk factors, precisely because the baseline risk of clinically significant VTE is so low [20]. The results obtained in this research bolster this recommendation that a typical Indian population of relatively young, low-risk patients, in whom no thrombotic events were observed, can be managed with basic preventive measures.

On the other hand, guidelines and studies emphasize prophylaxis in high-risk patients even if the surgery is laparoscopic. Garg et al. concluded that patients with advanced age, obesity, or prolonged operative time should receive thromboprophylaxis, as they observed greater coagulation activation in those subgroups [5]. This was in alignment with the risk-stratified approach of these research findings, with only three patients being classified as high-risk, and all of them were managed prophylactically, possibly averting potential clots. The incidence of post-operative DVT in the study group was one participant, consistent with numerous other studies showing rare venous thromboembolism events after LC in low-risk groups [21].

Limitations

The study cohort was predominantly male; hence, the results cannot be confidently generalized to females, as the coagulant status might differ in males. This gender imbalance limits the applicability of the findings of this research to the broader patient population, especially since cholecystectomy is also common in females. Lack of long-term follow-up was also a limitation for this research to study long-term complications, such as DVT.

## Conclusions

The findings of this laparoscopic cholecystectomy with CO₂ pneumoperitoneum induce subtle but significant coagulation changes indicative of a mild hypercoagulable state. Routine lab parameters like PT and INR are mainly reported within normal limits after laparoscopic cholecystectomy and might need additional markers for the detection of hypercoagulability. Additionally, markers like D-dimer can help reveal underlying prothrombotic shifts and prevent any associated adverse outcomes.
